# Extinction Debt in Source-Sink Metacommunities

**DOI:** 10.1371/journal.pone.0017567

**Published:** 2011-03-08

**Authors:** Nicolas Mouquet, Birte Matthiessen, Tom Miller, Andrew Gonzalez

**Affiliations:** 1 Institut des Sciences de l'Evolution - Centre National de la Recherche Scientifique - Université Montpellier 2. Place Eugène Bataillon, Montpellier, France; 2 Marine Ecology, Leibniz-Institute of Marine Science, Kiel, Germany; 3 Department of Biological Science, Florida State University, Tallahassee, Florida, United States of America; 4 Department of Biology, McGill University, Montréal, Canada; Dalhousie University, Canada

## Abstract

In an increasingly modified world, understanding and predicting the consequences of landscape alteration on biodiversity is a challenge for ecologists. To this end, metacommunity theory has developed to better understand the complexity of local and regional interactions that occur across larger landscapes. While metacommunity ecology has now provided several alternative models of species coexistence at different spatial scales, predictions regarding the consequences of landscape alteration have been done exclusively for the competition-colonization trade off model (CC). In this paper we investigate the effects of landscape perturbation on source-sink metacommunities. We show that habitat destruction perturbs the equilibria among species competitive effects within the metacommunity, driving both direct extinctions and an indirect extinction debt. As in CC models, we found a time lag for extinction following habitat destruction that varied in length depending upon the relative importance of direct and indirect effects. However, in contrast to CC models, we found that the less competitive species are more affected by habitat destruction. The best competitors can sometimes even be positively affected by habitat destruction, which corresponds well with the results of field studies. Our results are complementary to those results found in CC models of metacommunity dynamics. From a conservation perspective, our results illustrate that landscape alteration jeopardizes species coexistence in patchy landscapes through complex indirect effects and delayed extinctions patterns.

## Introduction

Habitat destruction and transformation is the dominant cause of biodiversity loss [1], [2], [3], [4], [5], [6], [7]. A substantial research effort is focused on understanding how habitat destruction modifies community structure and function [8], but understanding the extent and rate of species extinction due to habitat loss remains a challenge. Here we extend the application of metacommunity theory to the problem of extinction and provide new results for the rate and extent of species loss in fragmented landscapes.

Extensive habitat loss typically results in a mosaic of remnant fragments containing an area-specific subset of the flora and fauna. Habitat loss increases rates of local extinction due to a combination of direct effects associated with the loss of habitat, and the subsequent indirect effects due to habitat fragmentation and isolation that collectively initiate a process of community change in the remaining habitat fragments. The direct effects of habitat loss can involve the loss of critically important ‘source’ habitat that significantly reduces the metapopulation capacity of the landscape [9]. In a metacommunity context [10], indirect effects can cause extinction when habitat loss alters the pattern of species interactions that affect coexistence both locally [11] and regionally [12], [13].

Because habitat destruction can cause direct and indirect effects, species loss in remaining fragments is never immediate and, depending upon the degree of fragmentation, can involve a significant time delay (relaxation time as defined in [14]). That is, there will be a period after habitat fragmentation when community change has not occurred and the number of species present in the fragments is greater than the ultimate end state. Between the start and end of this disassembly process there is an extinction debt equal to the difference between the present and final species richness. Empirical support for this process comes from studies of oceanic islands following sea-level change [15], field surveys in terrestrial ecosystems [7], [16] and experiments with natural model systems [17].

Theory has also contributed to our understanding of the dynamics of extinction in fragmented landscapes. Although extinction is an assumed mechanism of island biogeography theory [18], recent theory with metapopulation [9], [19] and metacommunity models [13], [20] has explored how extinction occurs in spatially structured regions. Predictions from metacommunity theory stem predominantly from the competition-colonization (CC) trade off model of community coexistence [21], [22]. In CC models, poor competitors persist in the metacommunity because they have better colonizing ability than good competitors. In this case, patch habitat destruction lowers the colonization rate of all species but it has a greater effect on better competitors that have lower intrinsic colonizing ability [13], [23], [24]. Counter intuitively CC models predict that removing patches can result in an increase in the abundances of inferior competitors within the metacommunity and the slow extinction of superior competitors. Most significantly, due to the internal patch dynamics in this model, the subsequent species extinction is delayed [13]. Despite the obvious applied importance of community disassembly, the generality of the phenomenon has not been explored in other metacommunity models [25].

Source-sink metacommunity theory [12], [26] assumes that species persist locally and regionally in part through dispersal from source to sink habitats. As defined in Mouquet and Loreau [12], in the source-sink metacommunity model, coexistence is obtained through a compensation of differences in local competitive abilities at the scale of the metacommunity. This imposes a constraint on the distribution of species competitive abilities at the regional scale (called regional similarity). Generally this theory predicts that species richness at local and regional scales is maximal at intermediate levels of dispersal and environmental heterogeneity between communities [12], [27]. Source-sink dynamics are known to be important for species persistence in patchy and fragmented landscapes [28], [29], [30], [31] and often underlie the motivation for habitat corridors (e.g. [32]) and reserve design (e.g. [33]). Despite theoretical and empirical evidence for the prevalence of source-sink dynamics, virtually no predictions are available as to how habitat destruction and fragmentation will drive diversity loss in a source-sink system (but see [25]). Here we report a theoretical analysis of extinction dynamics in a source-sink metacommunity undergoing habitat destruction. By controlling dispersal and species relative performances we show how habitat loss mediates extinction. We find that habitat destruction drives extinction in two ways: (1) through a direct effect of habitat loss that removes critical source patches and has the greatest effect on habitat specialists, and (2) an indirect effect of habitat loss that disrupts competitive coexistence at the regional scale, causing both local and regional extinctions.

## Methods

### Source-sink metacommunity model

We modified the model of Mouquet and Loreau [12] that describes lottery competition between species within communities and migration among communities within a metacommunity. At the local scale, *P_ik_* is the proportion of micro-sites that can be occupied by only one individual of species *i* in community *k*. The metacommunity consists of *N* communities that differ in their local conditions where *S* species compete for a limited proportion of vacant microsites 
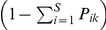
 . Each species *i* is characterized by a set of reproduction parameters, *c_ik_*, each of which defines the potential local reproductive rate of species *i* in community *k*, and a set of mortality rates, *m_ik_*. The distribution of parameters is such that each species potentially exhibits different reproductive rates in the different communities.

At the regional scale, the model assumes a constant proportion of dispersal among communities, *a*, equal for all species in all communities. Emigrants are equally redistributed to all other communities; except that no individuals return to the community from which they originate. We thus make a rather standard assumption about dispersal: that individuals disperse away at the risk of landing in a site where they are less well adapted and risk being competitively inferior. The equations read:
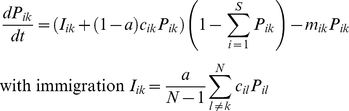
(1)


Without dispersal among communities, the species with the highest local basic reproductive rate (*r_ik_ = c_ik_/m_ik_*) excludes all other species in the local community. If, however, communities are linked by dispersal, and different species dominate in different communities (e.g., due to spatial heterogeneity in competitive rankings), local coexistence is possible. Individuals emigrating from source areas prevent competitive exclusion in sink areas (where they are competitively inferior). This situation has been called regional similarity (Mouquet and Loreau [11]) since it implies equivalence among regional competitive abilities between coexisting species.

### Simulations

In our simulation we have varied dispersal and the degree of regional similarity to generate different metacommunities [12]. For each metacommunity we randomly removed some communities to simulate habitat destruction and measured the consequences for species richness.

We considered the mortality rates equal (*m_ik_* = *m*) across species and based the competitive hierarchy only on potential local reproductive rate *c_ik_* (species *i* in community *k*). To generate the distribution of species parameters we have generated two types of matrices of *c_ik_* parameters. One matrix (called *Rand*) in which *c* values were randomly chosen between 0 and a maximal value *c_max_*. The other matrix (called *SRS*) fitting the constraint of strict regional similarity (as defined in Mouquet and Loreau [11]): each species has its *c_ik_ = c_max_*+*m* in one of the communities and the other parameters were derived such that in each community:
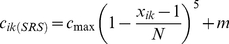
(2)with *N* the number of species, *x_ik_* the competitive rank of species *i* in community *k* and *m* is the mortality rate (we add *m* such that no species will have a negative potential reproductive rate in any of the communities). The exponent 5 makes the local competitive hierarchy relatively steep. The competitive ranks *x_ik_* are chosen so that each species is the best competitor in one community, the second best competitor in another community, the third in a third, etc. We then simulated metacommunities with different level of regional similarity by combining the matrices *Rand* and *SRS* in different proportions:
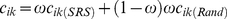
(3)with *ω* (varying between 0 and 1) defining the degree of regional similarity between species. This results in a set of competitive parameters ranging from strict regional similarity (*ω* = 1) to entirely random matrices (*ω* = 0).

In all our simulations, the mortality parameter *m* was fixed at 0.2 and the maximal reproductive rate *c_max_* at 5.0. Each metacommunity consisted of 20 species and 20 communities and species growth was simulated using an Euler approximation (Δt = 0.1). Each simulation was run for 100000 iterations, which allowed an equilibrium to be reached in all communities. At the beginning of each simulation, we attributed the same proportion of sites to each species in all communities (*P_ik_* = 0.01, for all *i* and *k*). To approximate stochastic extinction, we defined a species as extinct when its proportion of occupied sites was lower than an extinction threshold = 0.01 (after a period of time corresponding to the very early stage of community assembly, 2000 iterations).

Patch destruction was modeled by removing communities from the metacommunity at equilibrium and then measuring the effect on species richness. After sufficient time for an equilibrium to be reached (100000 iterations), 4 communities were randomly eliminated from each metacommunity and the dynamics were continued with only the remaining 16 communities until sufficient time for new equilibrium to be reached (100000 iterations). The equilibrium local species richness before and after the perturbation was measured, as well as identity of the species that went extinct; extinct species that have lost their source communities were considered extinct because of a *direct effect*, all others because of an *indirect effect*. We defined the *net indirect effect* as the proportion of species lost because of the indirect vs. direct effects (number of species extinct due to the indirect effect/total number of species extinct).

To avoid any pseudoreplication or confounding effect of a given destruction configuration we performed each simulation with a different metacommunity (using a new rand matrix) and a different destruction configuration. We generated 2000 different metacommunities, and for each metacommunity we varied dispersal and regional similarity between 0 and 1 (with 0.05 increment) generating 800000 simulations in total. For each metacommunity and each regional similarity value, we defined as a source for a species the community where that species was the best competitor, i.e. the community where it would win the competition if there was no migration among local communities.

We recorded the relaxation time for each extinct species as the time between when habitat destruction occurred and when extinction occurred. *Mean relaxation time* was then computed for each simulation over all extinct species. We also recorded the regional competitive ability of the remaining species (at the end of the simulation) and of the species that went extinct through the indirect effect. It was calculated for each species as their mean reproductive value among the 16 remaining communities. Finally, we provide a robustness analysis of our results as supplementary information (Supporting Information File S1, Fig. S3, S4, S5, S6, S7, S8, S9, and S10).

## Results

**Figure 1:**
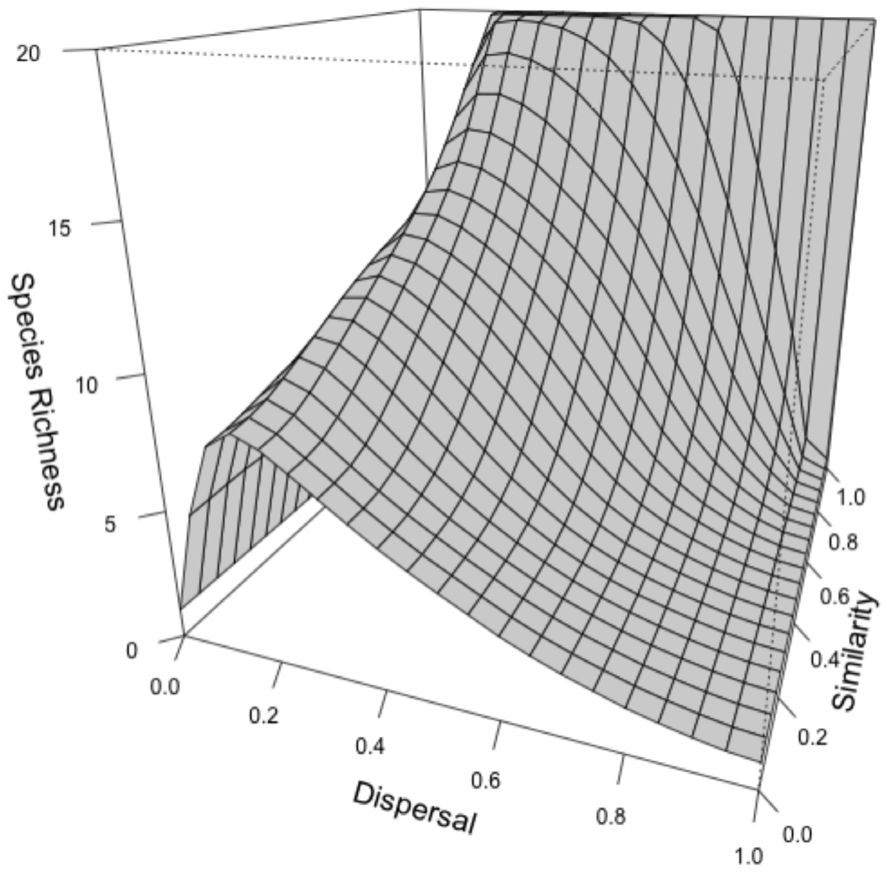
Mean local species richness in the metacommunity for 20 species and 20 communities, with increasing proportion of dispersal among communities and different values of regional similarity *ω* (from 0 to 1 with 0.05 increments). Other parameters are given in the text. We present means of 2000 simulations with different random matrices (*Rand* as defined in the methods). For clarity, we have omitted the standards deviation (however, they were always <15% of the means).

We first explored the metacommunity dynamics with no habitat destruction. Simulations were run varying the proportion of dispersal between communities and the degree of regional similarity (Fig. 1). As has previously been shown [12], varying the proportion of dispersal between communities always results in a positive unimodal relationship with local species richness (except when regional similarity is maximal). Increasing regional similarity shifts the peak to the right and increases the range of dispersal values over which species richness is maximal; the greatest species richness is attained when regional similarity is most strict.

### Identifying two components of the extinction debt

**Figure 2:**
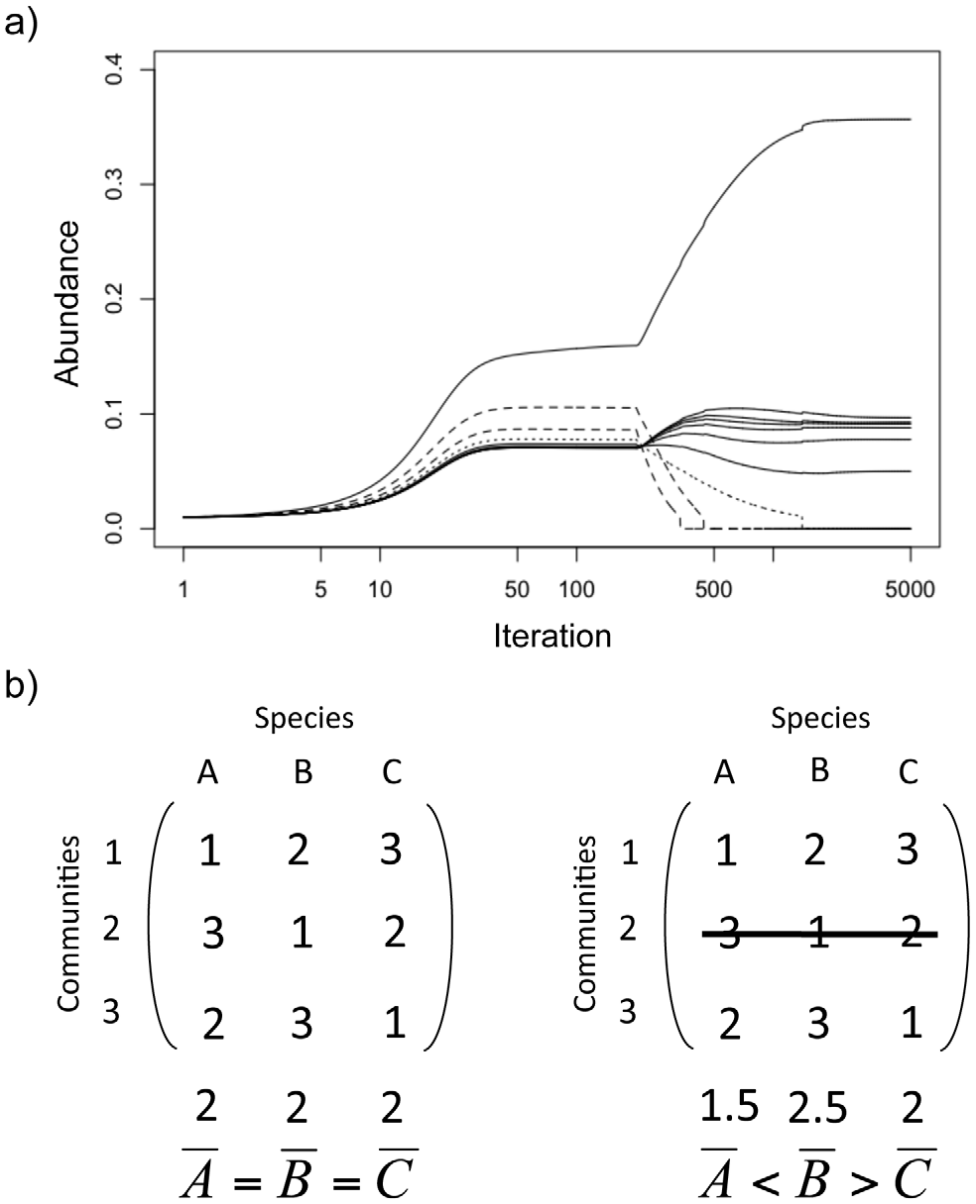
Direct and indirect consequences of habitat destruction. (a) Example of the consequence of habitat destruction on species dynamics within one community included in a metacommunity. For clarity we have simulated a metacommunity with only 10 species and 10 communities. Parameters are as described in the methods with *ω* = 1 and *a* = 0.7 (these values of *ω* and *a* were chosen to clearly illustrate extinction patterns). The simulation was run for 5000 iterations with a delta of 0.1 for the Euler approximation. We present species abundances (proportion of occupied sites) as a function of time (log scale). The destruction of two communities was simulated when equilibrium was reached (here after 200 iterations). The dashed lines represent the species lost through the direct effect and the dotted line the species lost through the indirect effect (see text). (b) A simple example of how the destruction of some communities from the metacommunity will alter the complimentarity in species' competitive ability and decrease the level of regional similarity. The figure gives a hypothetical distribution of competitive abilities in a metacommunity consisting of three species (A, B and C) that occur across three communities (1, 2 and 3). Averaging species competitive abilities at the scale of the region (line below the matrix) is the simplest definition of regional competitive ability. The left matrix illustrates the extreme case of strict regional similarity between competing species as defined in the text: each species is the best competitor in one community, but the species have equal (similar) competitive abilities at the scale of the region. In the right matrix we destroy one community from the metacommunity (community 2) and show how it leads to less similarity at the scale of the region. One species (species A) will be lost through the direct effect but another species (species C) can also be excluded from the metacommunity by the species (species B) that is now the best competitor at the scale of the region.

Figure 2a provides an illustration of the local dynamics that can follow habitat loss. In this example a metacommunity with 10 species in 10 communities was reduced to 8 communities after equilibrium was reached. Here the most obvious consequence of patch destruction was the loss of the two species that were specialists of the two communities destroyed (Fig. 2a, dashed lines). In the absence of their source community, these species cannot maintain a positive growth rate in the remaining communities. We have called this the “*direct component*” of the extinction because these extinctions are a direct consequence of losing source habitats.

However, figure 2a shows that one other species went extinct at a slower rate (dotted line). This additional extinction is due to the constraint of regional similarity. Coexistence in a source-sink metacommunity is possible if differences in local competitive abilities are compensated at the scale of the region through the appropriate distribution of species competitive abilities among communities (called regional similarity [11]). Destroying some communities from the metacommunity alters this spatial complementarity and results in less regional similarity between competing species (as illustrated in the Fig. 2b). This disruption of regional similarity is a consequence of habitat destruction and can lead to indirect competitive exclusion (see also Fig. 1 where lower regional similarity leads to lower species richness). We have called this secondary loss of species the “*indirect component*” of the extinction. The loss of species due to the indirect effect is slower than through the direct effect of habitat destruction (Fig. 2a). In addition, the distribution of abundances is also affected by habitat destruction, resulting in significant reorganization of relative abundance (Fig. 2a), but we will focus here on species loss.

**Figure 3:**
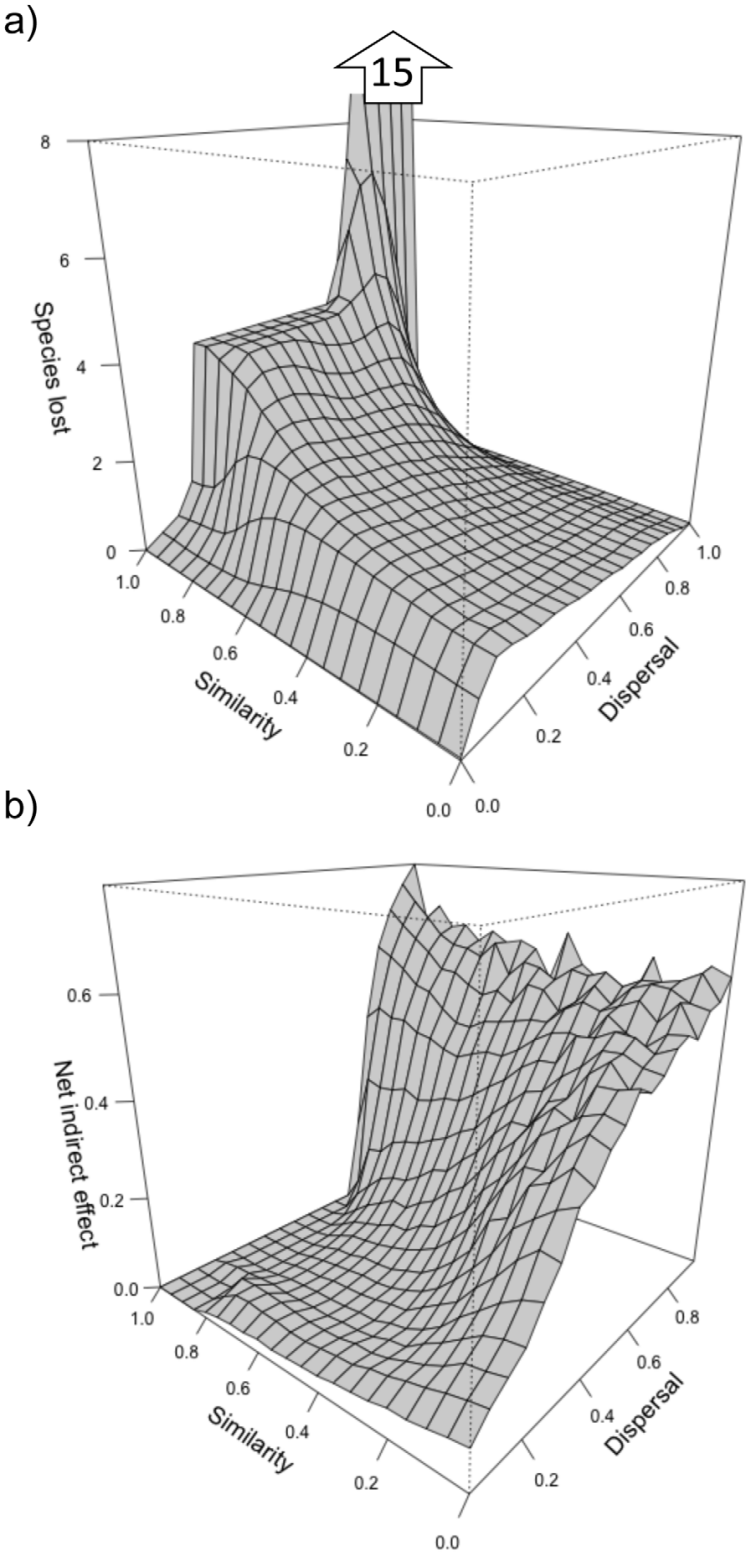
Effect of habitat destruction fro difference values of dispersal and regional similarity. Consequences of destroying 4 communities in a metacommunity of 20 communities and 20 competing species for different values of dispersal and regional similarity. We present means of 2000 simulations as described in the methods and the Fig. 1. (a) Total number of species lost (both through direct and indirect effects). (b) Net indirect effect (proportion of species lost because of the indirect vs. direct effects). Note that the z axis of the panel (a) has been limited to 8 species but the values go up to 15 species for high levels of dispersal and similarity. Note also that on the panel b, for clarity, we have not represented the values obtained for dispersal = 0 and 1. When dispersal = 0 there is no source sink dynamics and thus no indirect effect is possible (direct effect is always maximal when extinction happens, except at very low regional similarity when the distinction between sources and sink is less trivial). When dispersal = 1 the metacommunity is homogenized, local diversity is equal to one and there is no extinction after habitat destruction.

We found that the effect of patch destruction (in terms of number of species lost) was strongest at intermediate values of dispersal when source sink dynamics were important in maintaining high levels of pre-destruction local species richness (Fig. 3a). This is particularly strong at high values of regional similarity where destroying one community moves the metacommunity far from the “favorable” initial distribution of species competitive abilities (as illustrated in Fig. 2b). This effect is less pronounced as the regional similarity decreases (Fig. 3a).

We found that the relative importance of the direct and the indirect effects varies with dispersal and regional similarity. The direct effect is dominant only at low to intermediate dispersal values and high regional similarity (Fig. 3b). It is indeed at these values that the potential for source-sink dynamics is maximal; most species have specific sources and maintain a presence in other communities through dispersal. In this case destroying a community will result in losing a source for a specific species and will lead to direct extinction.

### Extinction Order and Relaxation time

**Figure 4:**
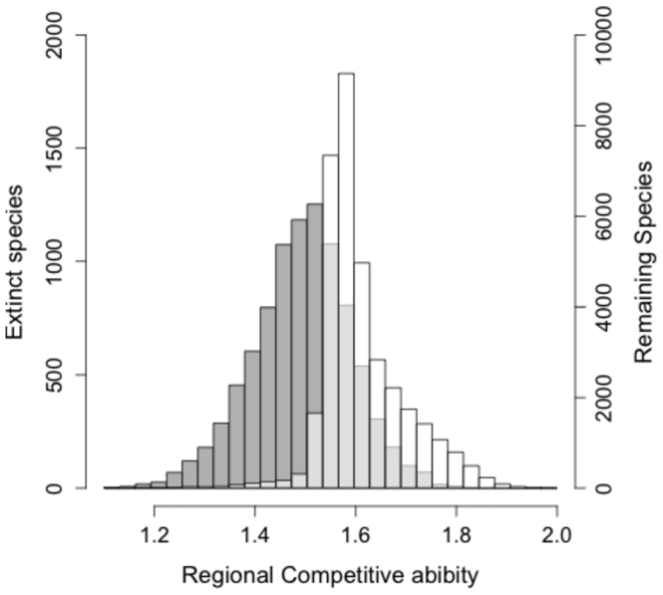
Regional Competitive abilities. Distribution of regional competitive abilities of the species extinct through the indirect effect (left axis, grey distribution) and the species remaining into the metacommunity at the end of each simulation (right axis, white distribution). Light grey indicates where the two distributions overlap. The results have been obtained by combining results found for the simulations presented in Figure 1 and 3 with fixed regional similarity *ω* = 0.8 and all dispersal values (between 0 and 1). Note that similar tendencies are found for other values of regional similarity (Fig. S1).

**Figure 5:**
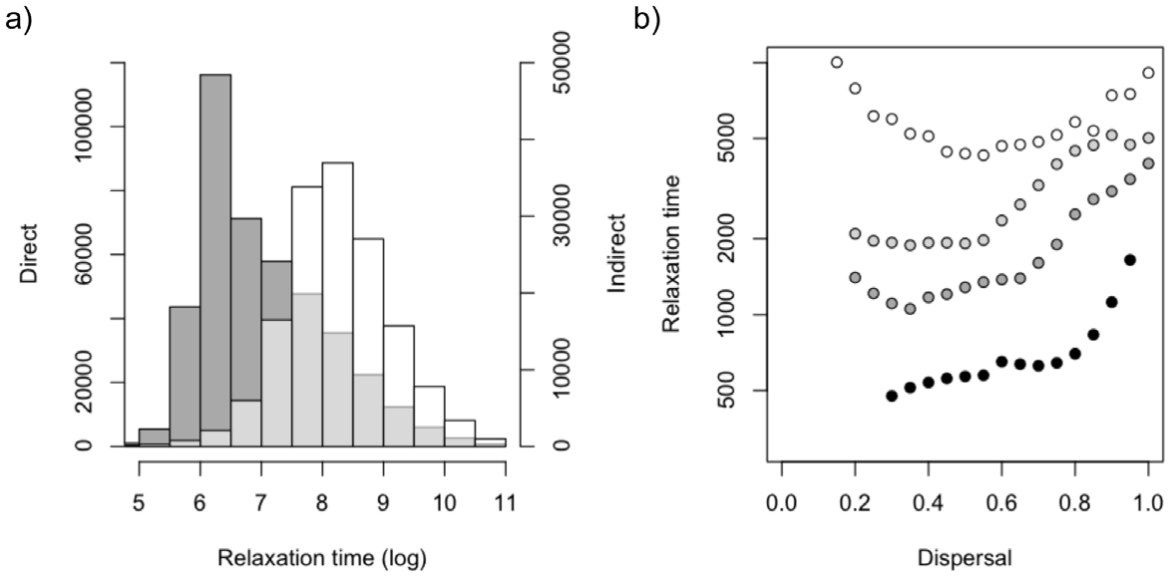
Relaxation time. (a) Distribution of the direct (left axes, grey distribution) and indirect (right axis, white distribution) values of relaxation time. We have combined all the values found obtained for all simulations (for all values of regional similarity and dispersal). Light grey has been used when the two distributions overlap.(b) Direct relaxation time with increasing dispersal and different values of regional similarity (*ω* = 0 white circles, *ω* = 0.5 light grey circles, *ω* = 0.7 dark grey circles, *ω* = 0.9 black circles). We present means calculated for 2000 simulations; parameters are given in methods. We do not present data where we found fewer than 30 simulations with regional extinctions (low values of dispersal). Standards deviations are omitted for clarity and are presented in Figure S2.

We found that the species that went extinct through the indirect effect were less competitive at the scale of the region than the remaining species in the metacommunity (Fig. 4). We also found that the time to extinction following community destruction was always longer through the indirect than the direct effect (Fig. 5). The degree of regional similarity influences the duration of the direct relaxation time. When regional similarity is high, losing a source has direct and rapid consequences on the species specialist on this source: the direct relaxation time is short (Fig. 5b). Moving away from regional similarity makes species less dependent on one particular source for their regional persistence and thus extinction, when it happens, takes longer. This tendency is more pronounced when dispersal is low (Fig. 5a, black circles). In most cases (Fig. 5a) direct relaxation time increases with dispersal, because the importance of individual sources in maintaining local species richness is less important as dispersal increases. This is, however, not true for very low regional similarity where the source-sink dynamics are also less important in maintaining local species richness. Regional similarity and dispersal have no clear pattern for the indirect effects (data not shown).

## Discussion

Metacommunity theory complements the significant contributions of island biogeography theory [18] and metapopulation theory [34] to deepen our understanding of the pressing problem of extinctions. We have extended the analysis of extinction in metacommunities to incorporate spatial heterogeneity in fitness and source-sink dynamics [12], [35]. We have found that habitat destruction (patch removal) has both direct and indirect negative effects on the magnitude and rate of local species loss.

### Direct and Indirect Effects of Habitat Loss on Extinction

By reducing the size of the metacommunity, habitat loss decreases the number of species that can coexist through the source-sink effect. With habitat heterogeneity and species sorting, each community within a metacommunity can support different sets of species. Losing communities also results in losing sources for some species, which is the equivalent of losing area in island biogeography models [18], [36]. In a metacommunity, a species that has lost its source will go extinct relatively quickly, which is the direct effect of habitat loss. However, we have shown that more complex indirect effects can occur as a consequence of the mechanism by which species coexist regionally in source-sink metacommunities. Even if species performances differ in each community, they may coexist within the metacommunity if their competitive abilities are equal when averaged at the scale of the region (“regional similarity” as defined in [12]). Removing some patches from the metacommunity makes species less regionally similar (as illustrated in Fig. 2b) and thus more species become prone to extinction than is expected simply from the loss of area; this is the indirect effect of habitat destruction on extinction.

In our model, the relative importance of direct and indirect effect change with dispersal and the regional competitive similarity. Mainly, the direct effects on extinction dominate when there are higher values of regional similarity (Fig. 3b); i.e., when the distribution of competitive abilities at the scale of the region is such that each species has a single unique community (source) in which it is strongly dominant. However, varying dispersal changes the (relative) importance of direct and indirect effects. That is, patch destruction leads to either extinction via the direct effect at low dispersal values or via the indirect effect at high dispersal values. From low to intermediate dispersal values, losing one source community means always losing the species that dominates in this source and thus the direct effect dominates. In less constrained situations with lower regional similarity, species richness is lower, the dynamics are no longer driven by the one species-one source situation and the indirect effects of habitat loss on extinction will be more important.

It is likely that in reality metacommunities are more complex than simple networks of identified sources and sinks and that the degree of regional similarity will not be very high. This makes the indirect effect more likely to be found in the field but also it makes predicting species loss following habitat destruction very difficult. Predictive power will only be gained through knowledge of regional as opposed to local performances. Classically indirect effects of fragmentation have been defined as a consequence of altered ecological interaction within the communities [1], [6], [37]. Here we have shown an indirect effect that can also arise by altering the spatial distribution of species interactions across a metacommunity (see also Mungia and Miller 2008).

### Extinction Order

One of the most important and yet controversial (see, for instance, [38]) results of the competition-colonization metacommunity model has been that the best competitors should go extinct first after habitat destruction [13]. In our source-sink metacommunity model, all species have equal dispersal abilities and thus the pattern of species extinction is not constrained by a trade off between colonization and competition (see also [25]). The best competitor at the scale of the region will thus have a lower probability of extinction because it has more communities acting as source habitat and thus is less affected by patch removal. We also found that some species might even increase in abundance after habitat destruction (Fig. 2a), because they increase their realized competitive abilities at the scale of the metacommunity (e.g., species B in the Fig. 2b). Thus, we conclude from our model that good competitors are likely to be less affected by habitat destruction than less competitive species, a result that corresponds well with the many studies where good competitors have been shown to be positively affected by habitat fragmentation (reviewed in [38]).

### Relaxation time

Another important result of the competition-colonization metacommunity model has been that it predicts that extinction will occur over a long time period following habitat destruction [13]. This result has been found in many different empirical studies, without necessarily any evidence for a trade off between competition and colonization [7], [16], [17], [38], [39], [40]. Our model also predicts that extinctions can occur with a delay after habitat loss and that the relaxation time will vary in length depending upon the relative importance of direct and indirect effects. In general we have found the indirect relaxation time is longer than the direct relaxation time (Fig. 5). Species lost through the indirect effect tend to have a source in the metacommunity, which delays their decline to extinction. When both effects occur in concert we predict that the relaxation time in natural systems will be characterized by two phases: an initial phase involving rapid extinction due to the direct effect, and a slower second phase involving a second bout of extinction due to the indirect effect. This prediction provides a novel expectation and guideline for future extinction analyses of time series data.

### Caveats and future work

Our model has considered one case of landscape alteration but other scenarios are possible, such as patch isolation or alteration of patch dynamics [4]. For instance, we have not considered the possibility of patch re-colonization after disturbance, as in the patch dynamics competition-colonization metacommunity model [20], [21]. We also have not considered the consequences of patch isolation because such effects have been already illustrated in a previous paper [35] (see also [4]) where it was shown that, counter intuitively, patch isolation could lead to positive effects on species richness when the metacommunity was highly connected. Removing habitat can also increase patch isolation in a spatially explicit context; removing a community from the metacommunity means also reducing dispersal between the adjacent communities [4]. Also in our model, the competitive hierarchy was based on varying the spatial distribution of the reproductive parameter while keeping mortality constant as in [12]. However, Muko and Iwasa [41] have found that the conditions for coexistence are less stringent when spatial heterogeneity in competitive abilities is obtained by varying mortality rather than the reproductive hierarchy, and dispersal is maximal. Finally, for simplicity we have restricted our analysis to particular combinations of species parameters and provide a robustness analysis as supplementary information (Supporting Information File S1, Fig. S3, S4, S5, S6, S7, S8, S9 and S10).

The source-sink framework is general enough to incorporate these additional complexities, and future research will address these issues as well as other important directions. A next step is to study the dynamics of extinction within more complex ecological situations by integrating trophic interactions [7], [42], [43], [44], [45], [46] and nutrient fluxes [47], [48] within the metacommunity perspective.

### Conclusion

We have analyzed the direct and indirect effects of habitat loss on extinction within source-sink metacommunities. Significant indirect extinction is a very likely outcome of patch destruction in the field because spatial variation in species competitive hierarchies within metacommunities is likely to be common. Discriminating between direct and indirect species extinction in the field is essential for understanding the causes of extinction and predicting the duration and timing of extinctions after habitat transformation. The most important message of this metacommunity model is that landscape alteration jeopardizes species coexistence in patchy landscapes through both the direct loss of source habitats and complex, often delayed, indirect effects. From a conservation perspective this reinforces the view that communities are embedded within a broader metacommunity context. Our approach has placed the study of extinction debts within a broader and more realistic community framework [6], [49].

## Supporting Information

Figure S1 Distribution of regional competitive abilities of the species extinct through the indirect effect (left axis, grey distribution) and the species remaining in the metacommunity at the end of each simulation (right axis, white distribution) for four different values of regional similarity (*ω* = 1, *ω* = 0.8, *ω* = 0.5, *ω* = 0). Other parameters and simulation method are as in figure 4.
(TIF)

Figure S2 Mean and standard deviations found for the direct relaxation time as presented in figure 5b for two values of regional similarity (a, *ω* = 0.5 and *b, ω* = 0.9). The standards deviations are high but the tendencies described in the text (that direct relaxation time increases with dispersal and decreases with regional similarity) hold. This is illustrated by comparing the distributions of relaxation time values (c,d) obtained for two values of dispersal (corresponding to the vertical dashed lines on the panel a and b) for each regional similarity scenarios (c, *ω* = 0.5 and *d, ω* = 0.9).
(TIF)

Figure S3 Mean of local species richness in the metacommunity for 20 species and 20 communities (method as described in Fig. 1). We performed 2000 simulations for three different values of theta (*θ* = 5 steep competitive hierarchy, *θ* = 1 linear competitive hierarchy and *θ* = 2.5 intermediate scenario).
(TIF)

Figure S4 Number of species lost (both through direct and indirect effects) and the net indirect effect (proportion of species lost because of the indirect vs. direct effects) with varying dispersal and regional similarity (method as described in Fig. 3). We performed 2000 simulations for three different values of theta (*θ* = 5 steep competitive hierarchy, *θ* = 1 linear competitive hierarchy and *θ* = 2.5 intermediate scenario).
(TIF)

Figure S5 Distribution of regional competitive abilities of the species extinct through the indirect effect (left axis, grey distribution) and the species remaining in the metacommunity at the end of each simulation (right axis, white distribution). Method as described in Fig. 4. We performed 2000 simulations for three different values of theta (*θ* = 5 steep competitive hierarchy, *θ* = 1 linear competitive hierarchy and *θ* = 2.5 intermediate scenario).
(TIF)

Figure S6 Distribution of the direct (left axes, grey distribution) and indirect (right axis, white distribution) values of relaxation time (method as described in Fig. 5a). And the direct relaxation time (method as described in Fig. 5b) with increasing dispersal and different values of regional similarity (*ω* = 0 white circles, *ω* = 0.5 light grey circles, *ω* = 0.7 dark grey circles, *ω* = 0.9 black circles). We performed 2000 simulations for three different values of theta (*θ* = 5 steep competitive hierarchy, *θ* = 1 linear competitive hierarchy and *θ* = 2.5 intermediate scenario).
(TIF)

Figure S7 Mean of local species richness in the metacommunity for 20 species and 20 communities (method as described in Fig. 1). We performed 2000 simulations for three different combinations of *c_max_* and *m* (*c_max_* = 5 and *m* = 0.2, *c_max_* = 2.5 and *m* = 0.2, *c_max_* = 5 and *m* = 1).
(TIF)

Figure S8 Number of species lost (both through direct and indirect effects) and the net indirect effect (proportion of species lost because of the indirect vs. direct effects) with varying dispersal and regional similarity (method as described in Fig. 3). We performed 2000 simulations for three different combinations of *c_max_* and *m* (*c_max_* = 5 and *m* = 0.2, *c_max_* = 2.5 and *m* = 0.2, *c_max_* = 5 and *m* = 1).
(TIF)

Figure S9 Distribution of regional competitive abilities of the species extinct through the indirect effect (left axis, grey distribution) and the species remaining in the metacommunity at the end of each simulation (right axis, white distribution). Method as described in Fig. 4. We performed 2000 simulations for three different combinations of *c_max_* and *m* (*c_max_* = 5 and *m* = 0.2, *c_max_* = 2.5 and *m* = 0.2, *c_max_* = 5 and *m* = 1).
(TIF)

Figure S10 Distribution of the direct (left axes, grey distribution) and indirect (right axis, white distribution) values of relaxation time (method as described in Fig. 5a). And the direct relaxation time (method as described in Fig. 5b) with increasing dispersal and different values of regional similarity (*ω* = 0 white circles, *ω* = 0.5 light grey circles, *ω* = 0.7 dark grey circles, *ω* = 0.9 black circles). We performed 2000 simulations for three different combinations of *c_max_* and *m* (*c_max_* = 5 and *m* = 0.2, *c_max_* = 2.5 and *m* = 0.2, *c_max_* = 5 and *m* = 1).
(TIF)

File S1 Robustness of our results to variation in (i) the steepness of competitive hierarchy and (ii) the maximal reproductive value (*c_max_*) and mortality (*m*).
(DOC)
