# Supplementary figures and images for: Extinction Debt in Source-Sink Metacommunities
Source: PLoS One. 2011 Mar 8;6(3):e17567. doi: 10.1371/journal.pone.0017567 (PMC3050922; doi:10.1371/journal.pone.0017567)

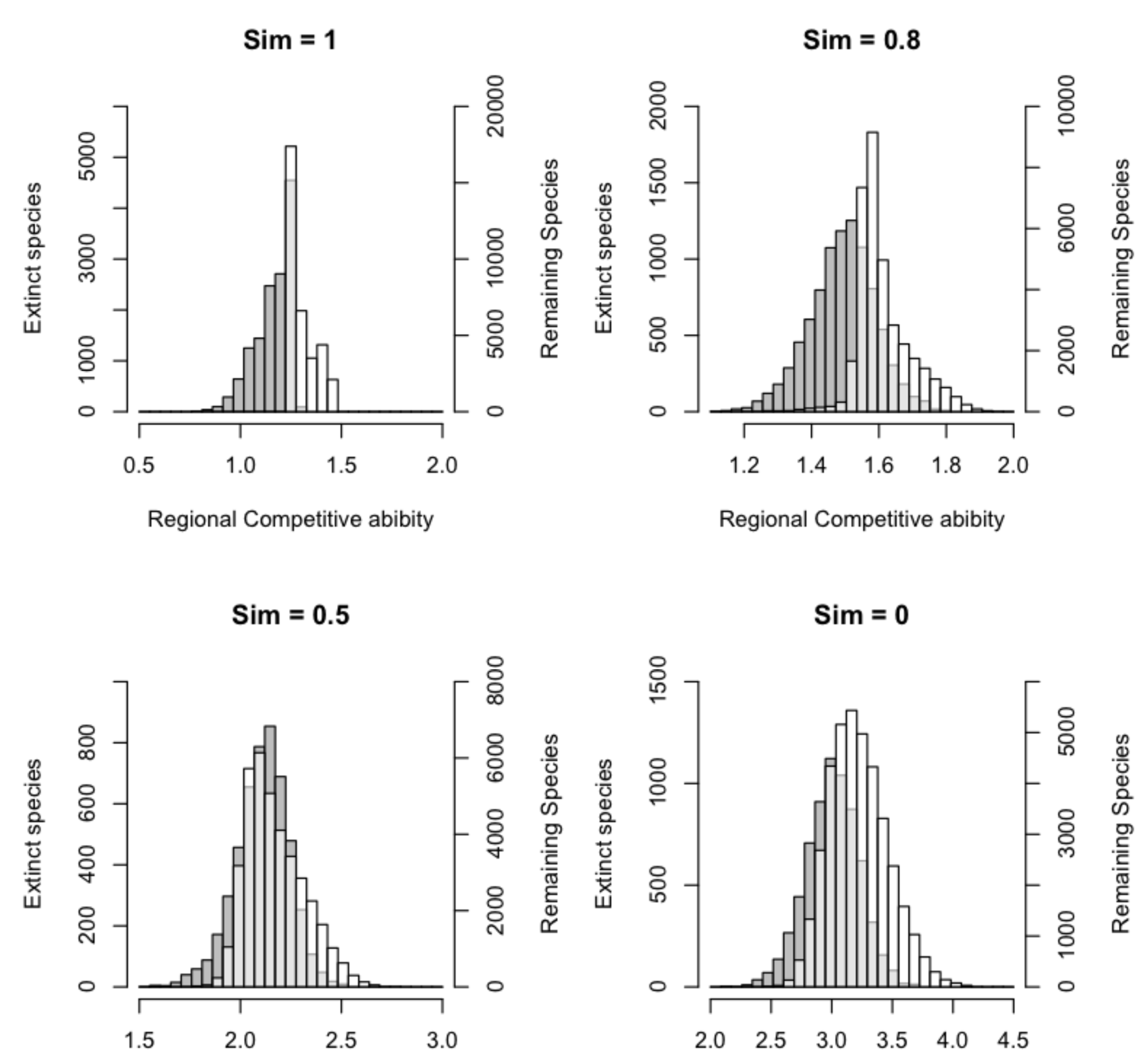

Supplement: Figure S1 — Distribution of regional competitive abilities of the species extinct through the indirect effect (left axis, grey distribution) and the species remaining in the metacommunity at the end of each simulation (right axis, white distribution) for four different values of regional similarity (ω = 1, ω = 0.8, ω = 0.5, ω = 0). Other parameters and simulation method are as in figure 4. (TIF) [file pone.0017567.s001.tif]

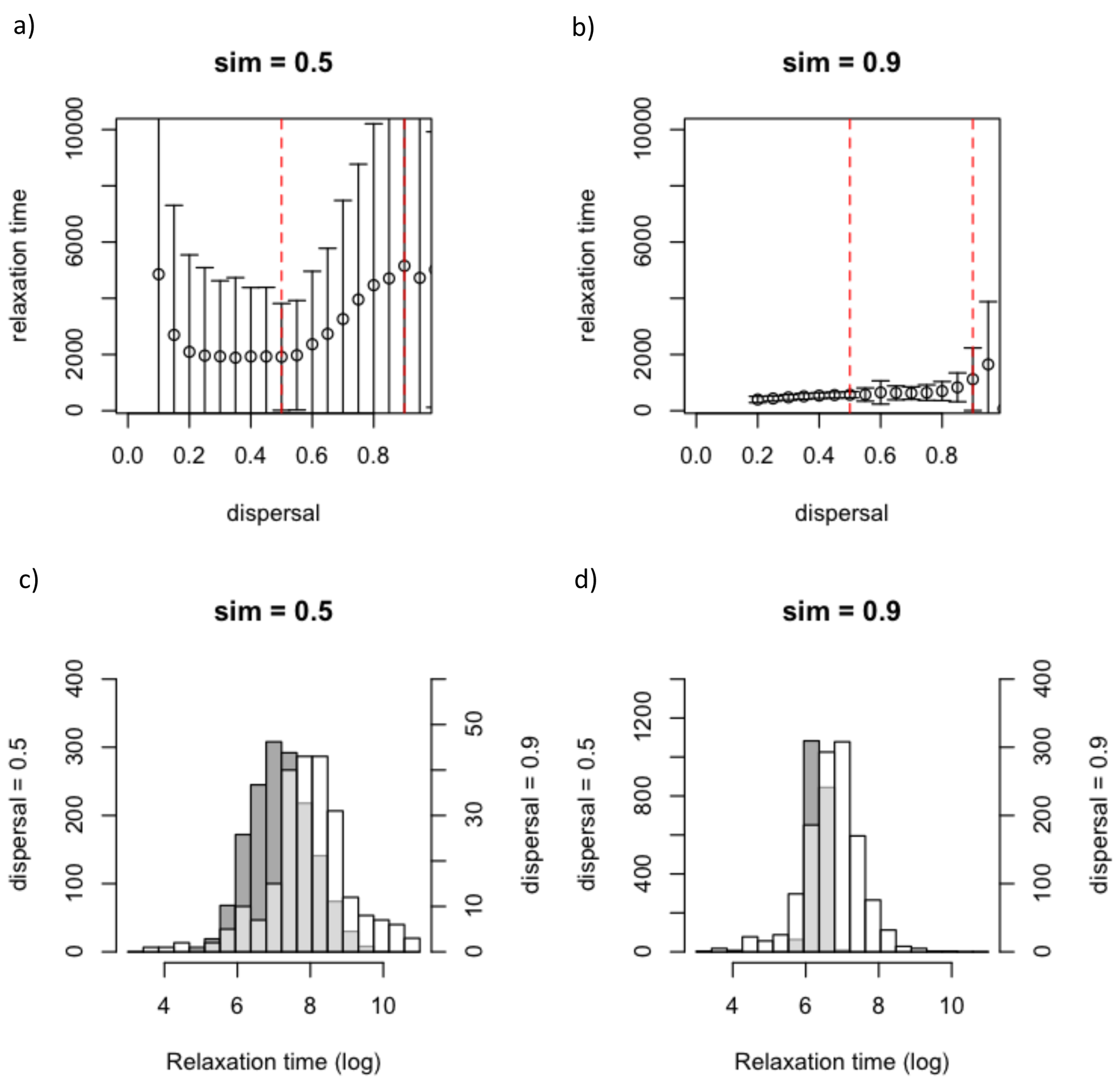

Supplement: Figure S2 — Mean and standard deviations found for the direct relaxation time as presented in figure 5b for two values of regional similarity (a, ω = 0.5 and b, ω = 0.9). The standards deviations are high but the tendencies described in the text (that direct relaxation time increases with dispersal and decreases with regional similarity) hold. This is illustrated by comparing the distributions of relaxation time values (c,d) obtained for two values of dispersal (corresponding to the vertical dashed lines on the panel a and b) for each regional similarity scenarios (c, ω = 0.5 and d, ω = 0.9). (TIF) [file pone.0017567.s002.tif]

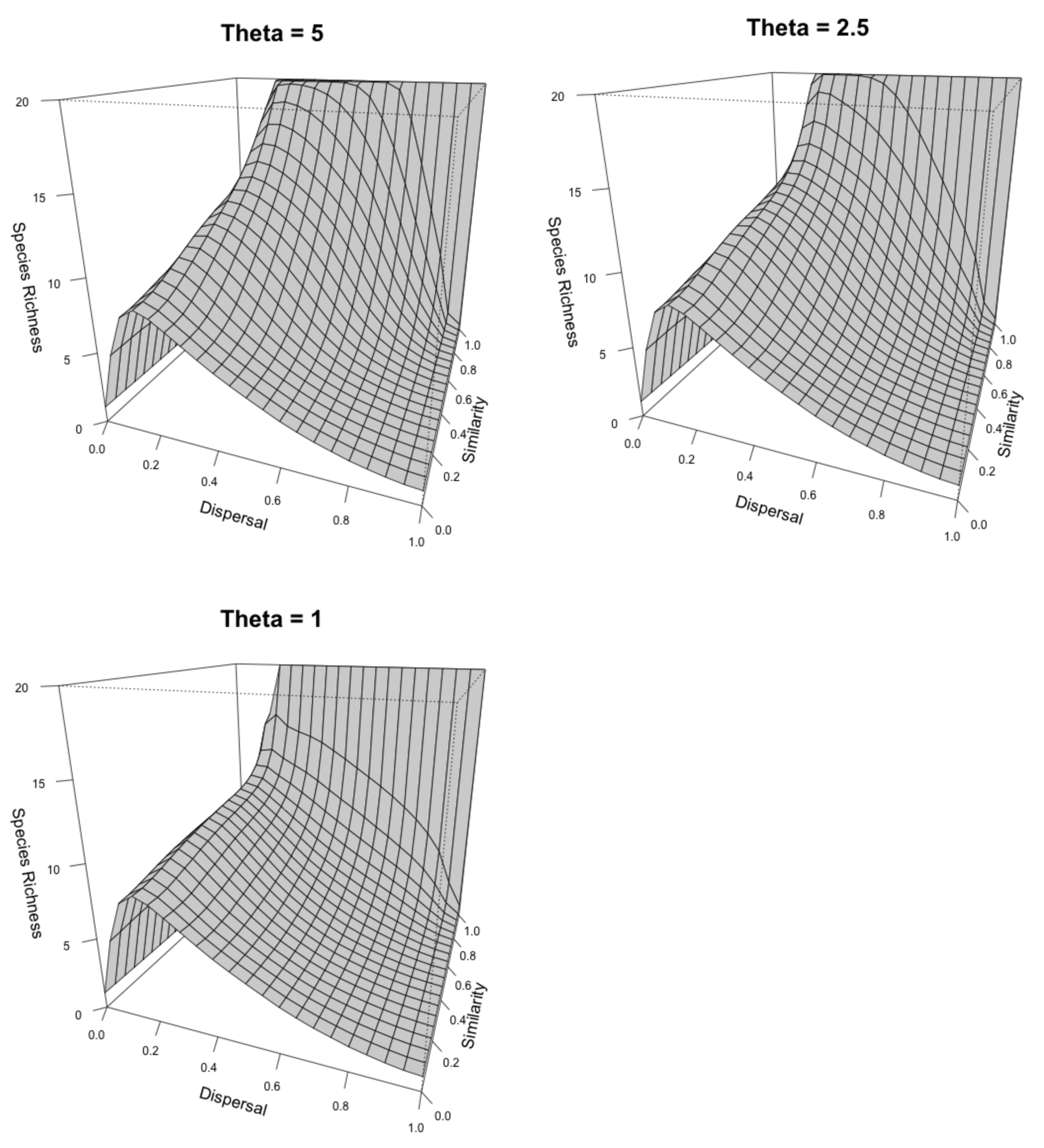

Supplement: Figure S3 — Mean of local species richness in the metacommunity for 20 species and 20 communities (method as described in Fig. 1). We performed 2000 simulations for three different values of theta (θ = 5 steep competitive hierarchy, θ = 1 linear competitive hierarchy and θ = 2.5 intermediate scenario). (TIF) [file pone.0017567.s003.tif]

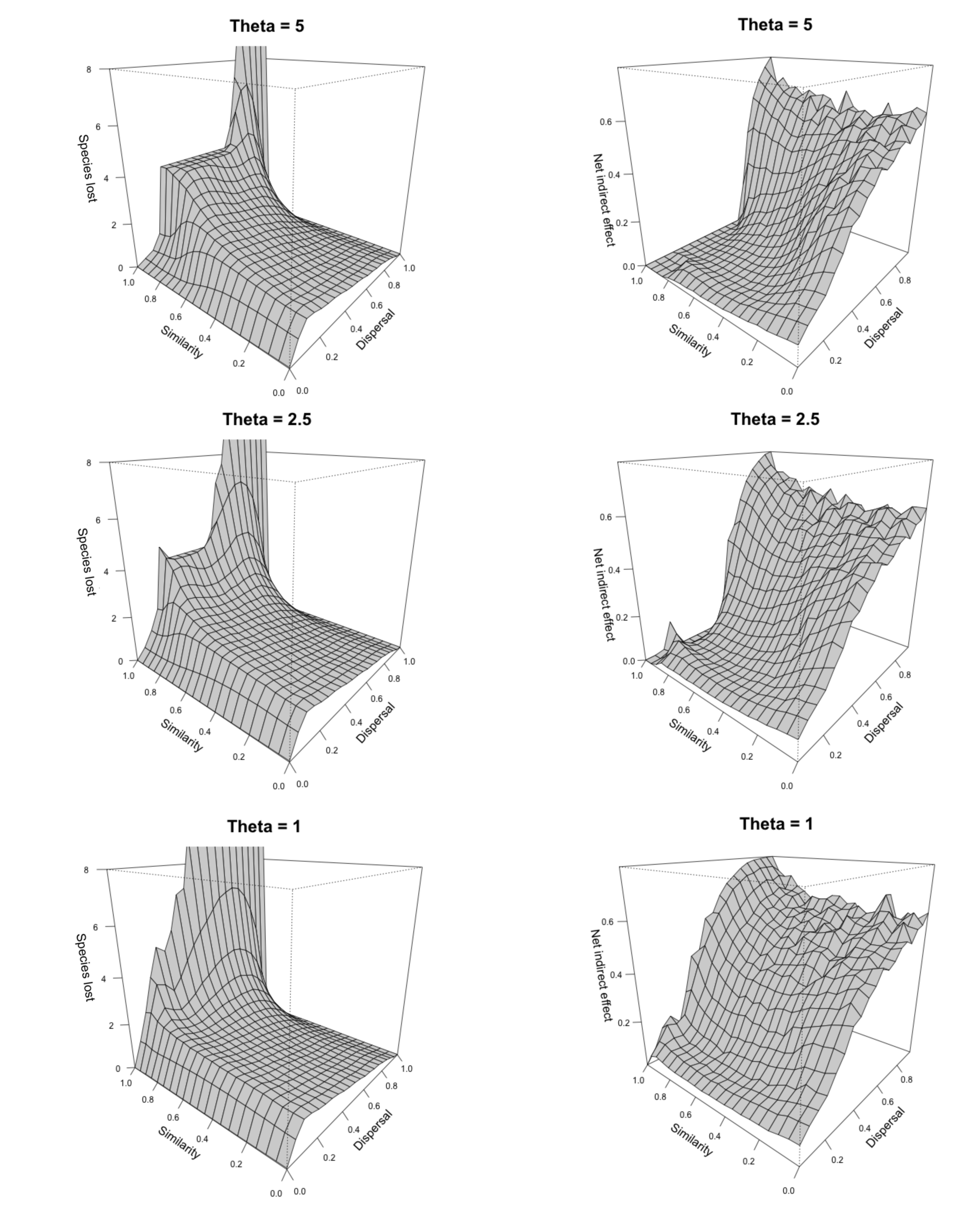

Supplement: Figure S4 — Number of species lost (both through direct and indirect effects) and the net indirect effect (proportion of species lost because of the indirect vs. direct effects) with varying dispersal and regional similarity (method as described in Fig. 3). We performed 2000 simulations for three different values of theta (θ = 5 steep competitive hierarchy, θ = 1 linear competitive hierarchy and θ = 2.5 intermediate scenario). (TIF) [file pone.0017567.s004.tif]

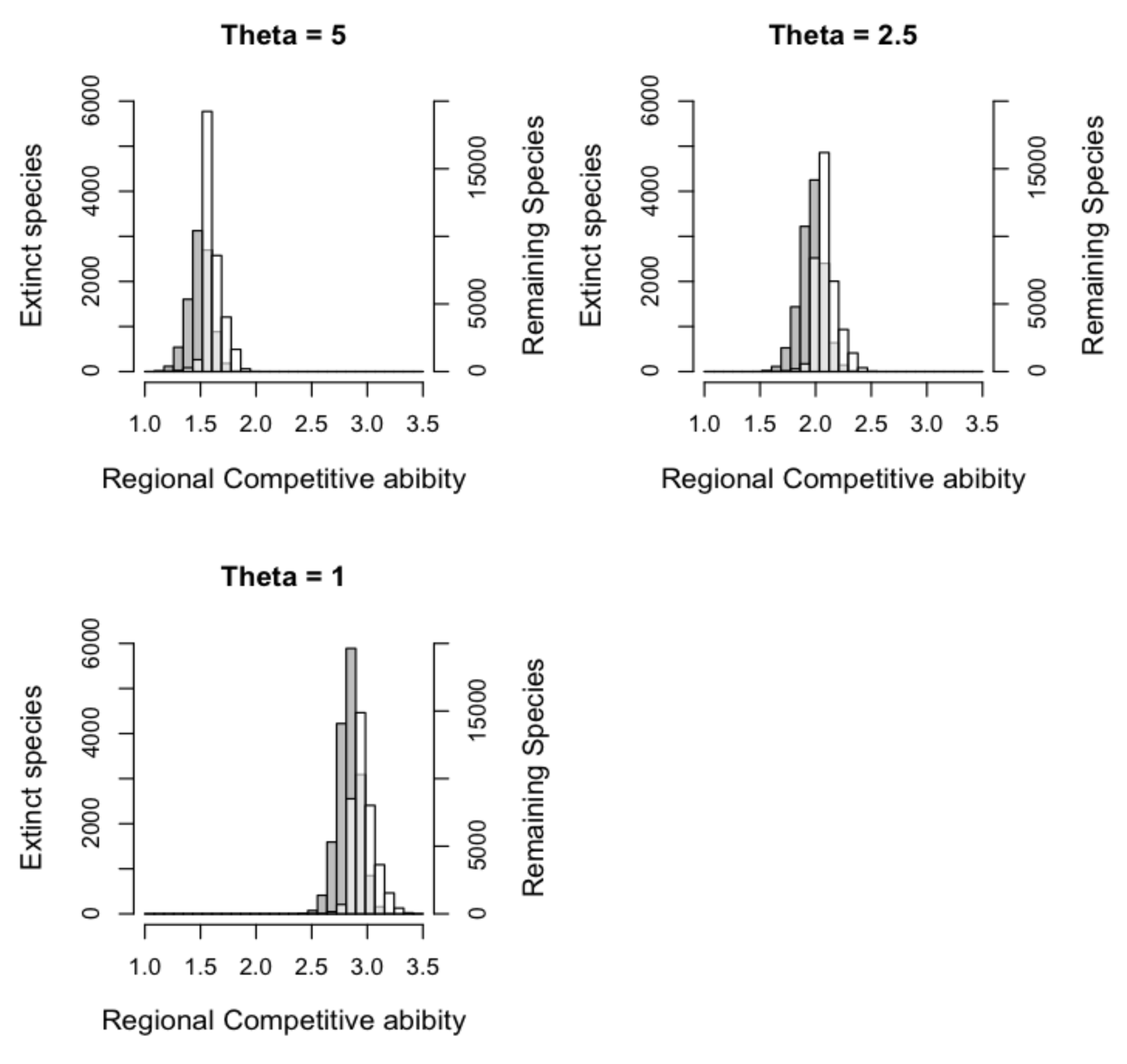

Supplement: Figure S5 — Distribution of regional competitive abilities of the species extinct through the indirect effect (left axis, grey distribution) and the species remaining in the metacommunity at the end of each simulation (right axis, white distribution). Method as described in Fig. 4. We performed 2000 simulations for three different values of theta (θ = 5 steep competitive hierarchy, θ = 1 linear competitive hierarchy and θ = 2.5 intermediate scenario). (TIF) [file pone.0017567.s005.tif]

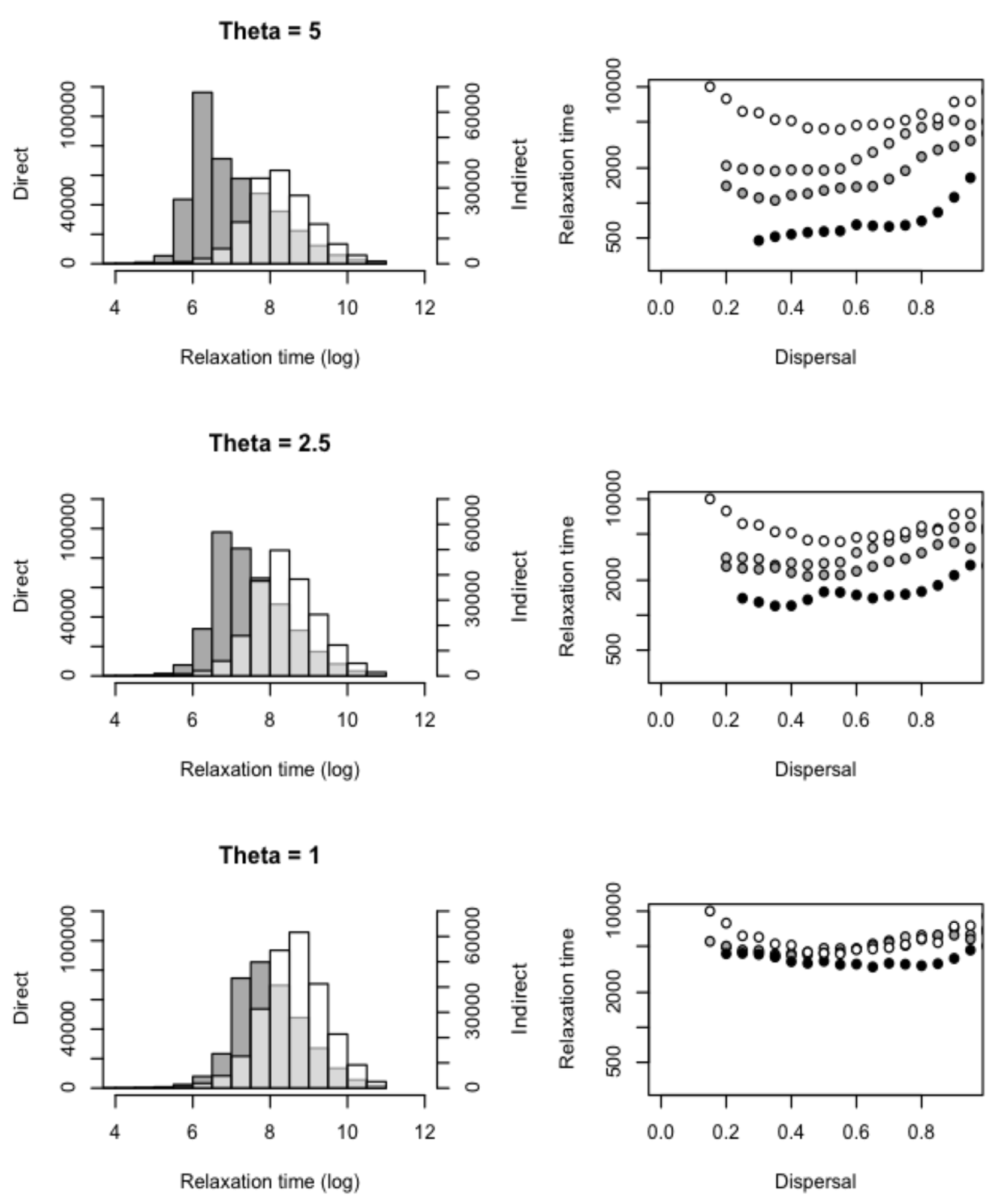

Supplement: Figure S6 — Distribution of the direct (left axes, grey distribution) and indirect (right axis, white distribution) values of relaxation time (method as described in Fig. 5a). And the direct relaxation time (method as described in Fig. 5b) with increasing dispersal and different values of regional similarity (ω = 0 white circles, ω = 0.5 light grey circles, ω = 0.7 dark grey circles, ω = 0.9 black circles). We performed 2000 simulations for three different values of theta (θ = 5 steep competitive hierarchy, θ = 1 linear competitive hierarchy and θ = 2.5 intermediate scenario). (TIF) [file pone.0017567.s006.tif]

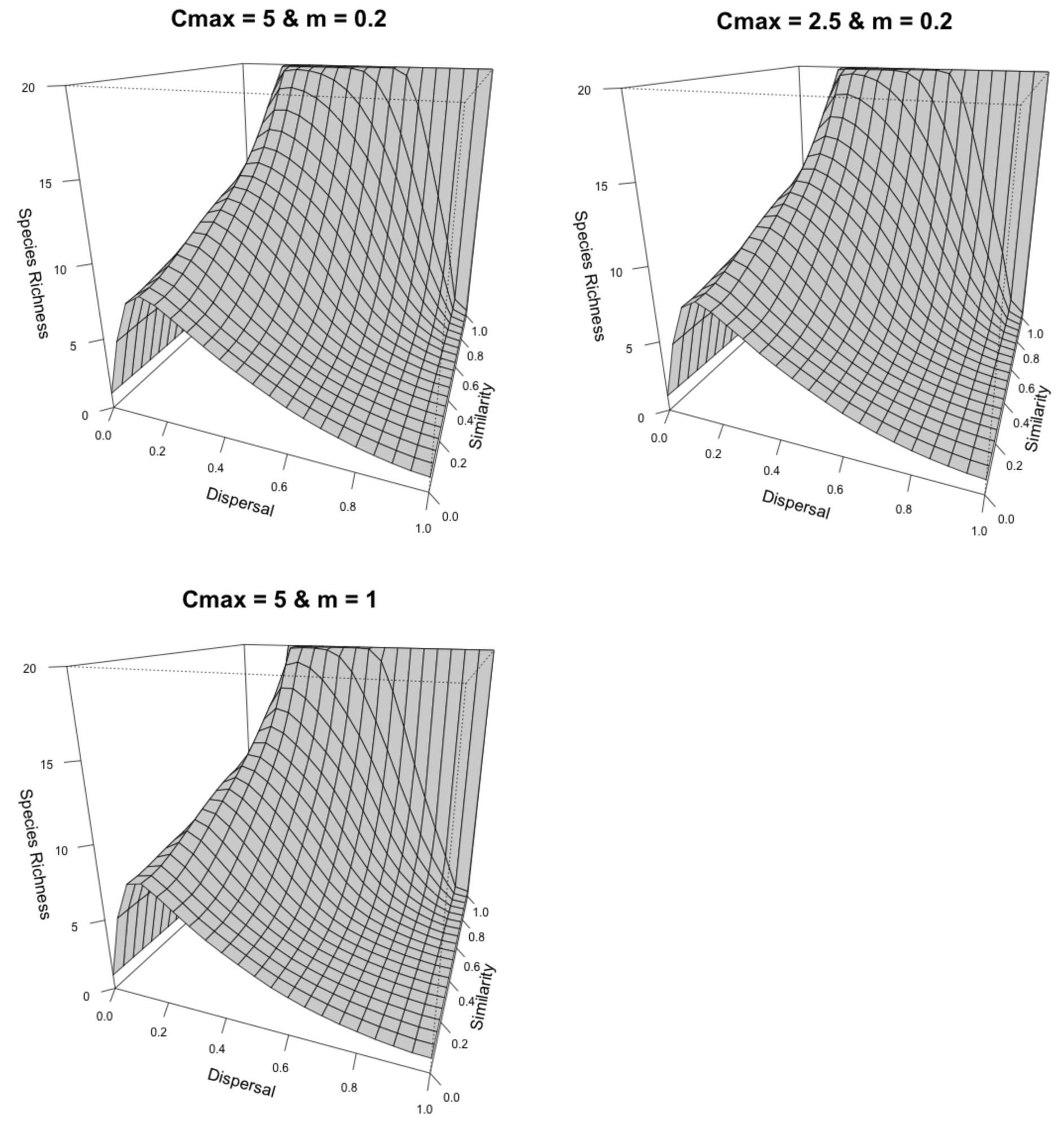

Supplement: Figure S7 — Mean of local species richness in the metacommunity for 20 species and 20 communities (method as described in Fig. 1). We performed 2000 simulations for three different combinations of cmax and m (cmax = 5 and m = 0.2, cmax = 2.5 and m = 0.2, cmax = 5 and m = 1). (TIF) [file pone.0017567.s007.tif]

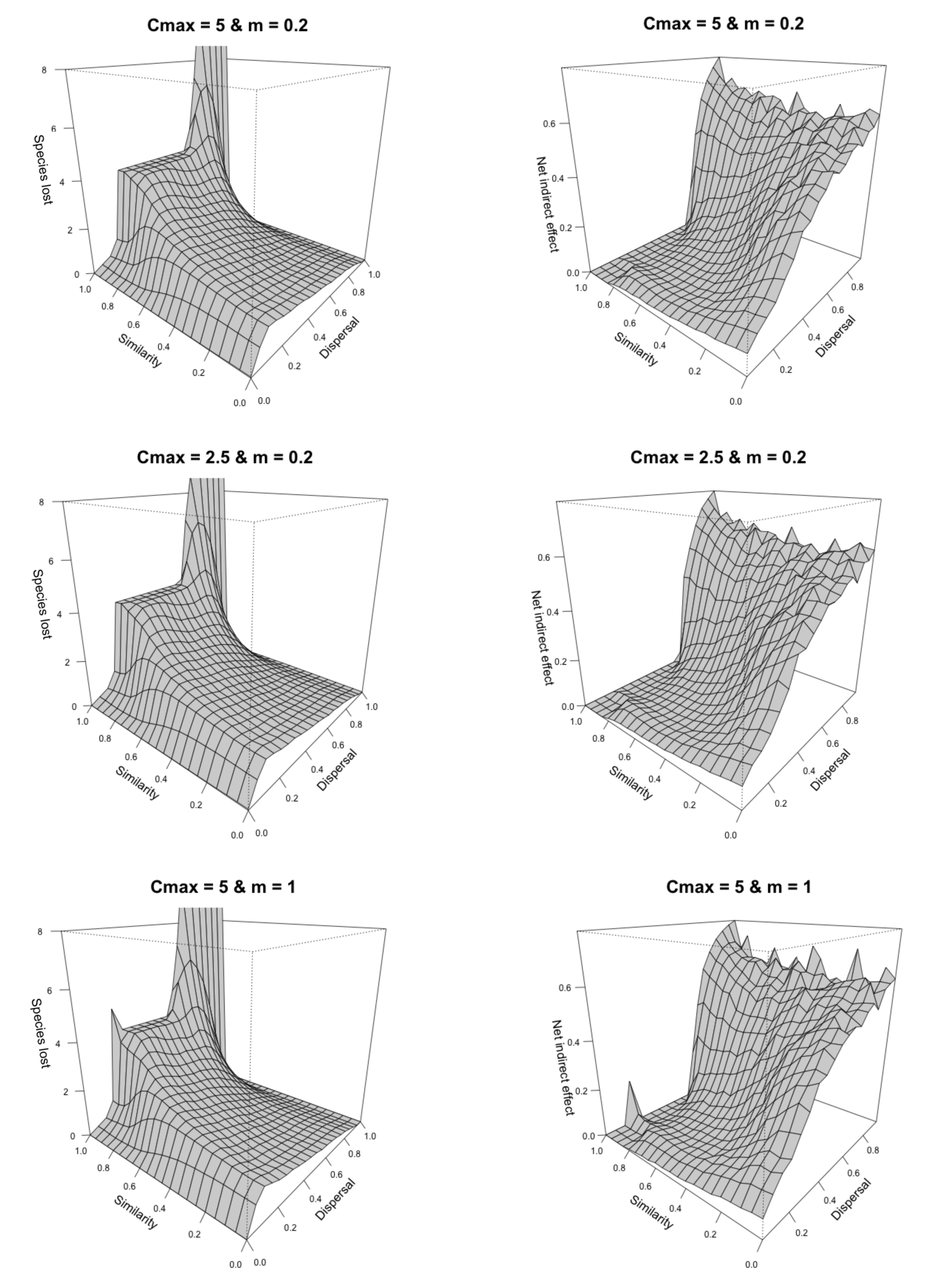

Supplement: Figure S8 — Number of species lost (both through direct and indirect effects) and the net indirect effect (proportion of species lost because of the indirect vs. direct effects) with varying dispersal and regional similarity (method as described in Fig. 3). We performed 2000 simulations for three different combinations of cmax and m (cmax = 5 and m = 0.2, cmax = 2.5 and m = 0.2, cmax = 5 and m = 1). (TIF) [file pone.0017567.s008.tif]

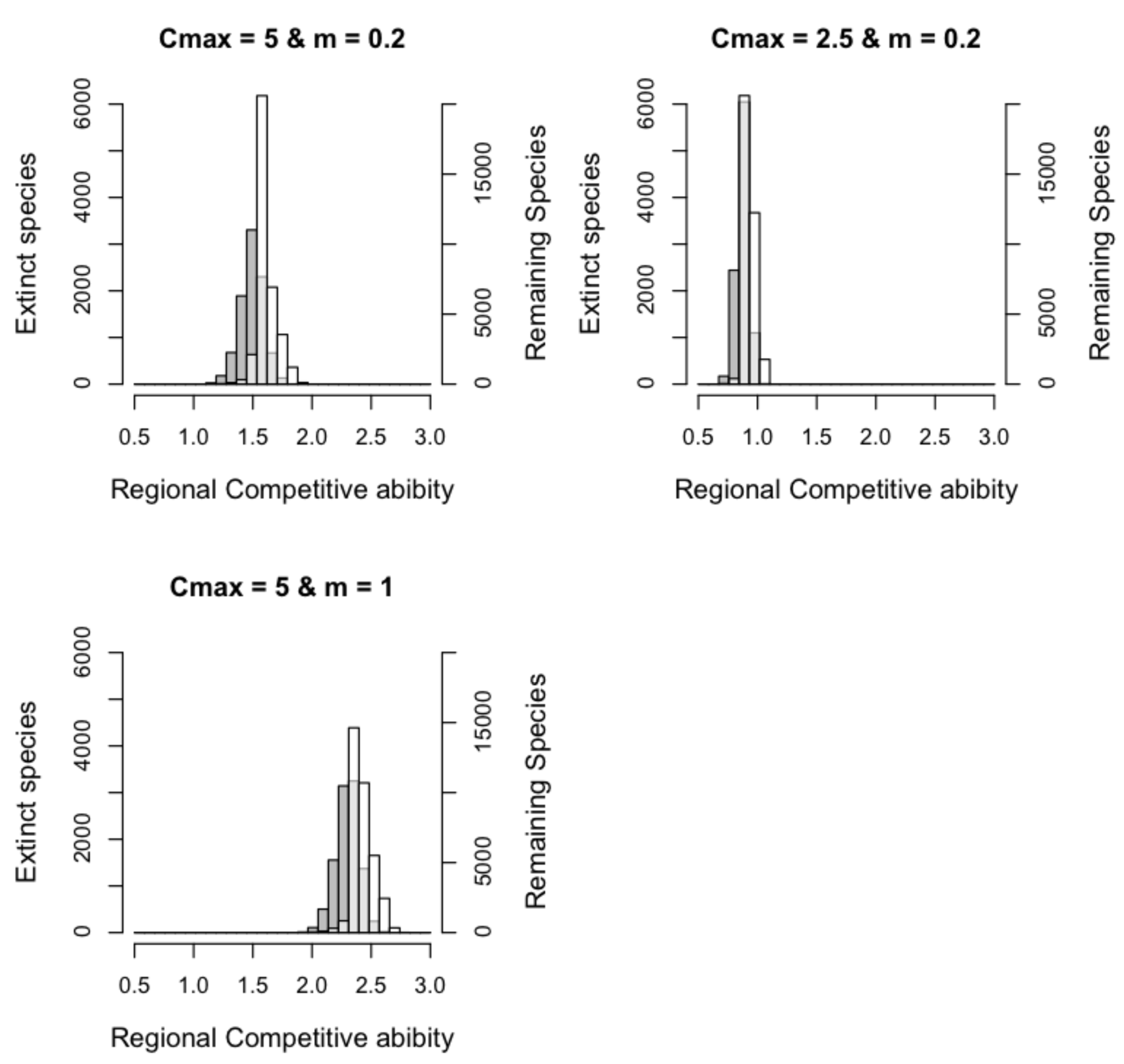

Supplement: Figure S9 — Distribution of regional competitive abilities of the species extinct through the indirect effect (left axis, grey distribution) and the species remaining in the metacommunity at the end of each simulation (right axis, white distribution). Method as described in Fig. 4. We performed 2000 simulations for three different combinations of cmax and m (cmax = 5 and m = 0.2, cmax = 2.5 and m = 0.2, cmax = 5 and m = 1). (TIF) [file pone.0017567.s009.tif]

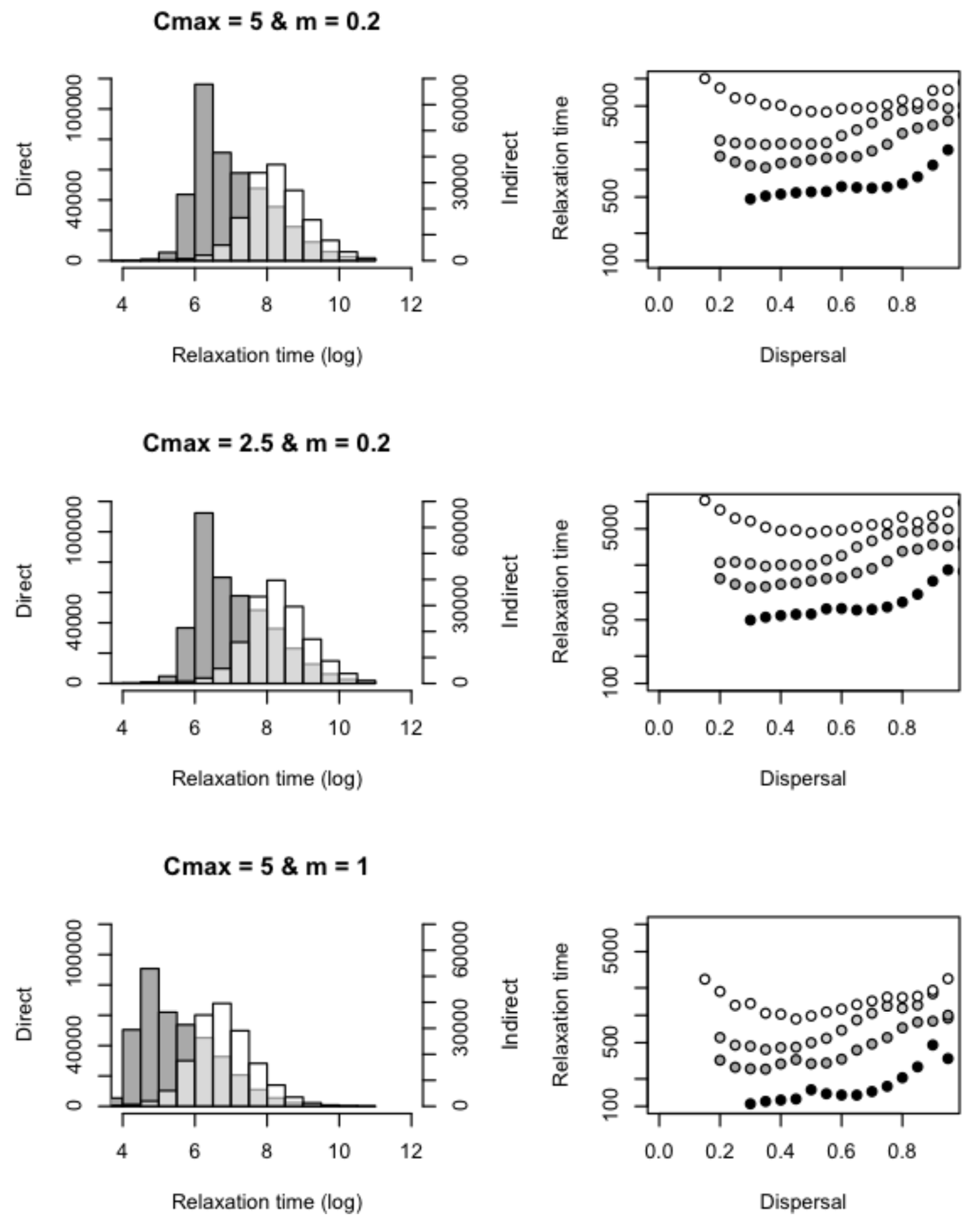

Supplement: Figure S10 — Distribution of the direct (left axes, grey distribution) and indirect (right axis, white distribution) values of relaxation time (method as described in Fig. 5a). And the direct relaxation time (method as described in Fig. 5b) with increasing dispersal and different values of regional similarity (ω = 0 white circles, ω = 0.5 light grey circles, ω = 0.7 dark grey circles, ω = 0.9 black circles). We performed 2000 simulations for three different combinations of cmax and m (cmax = 5 and m = 0.2, cmax = 2.5 and m = 0.2, cmax = 5 and m = 1). (TIF) [file pone.0017567.s010.tif]
