# Supplementary material for: Extinction Debt in Source-Sink Metacommunities
Source: PLoS One. 2011 Mar 8;6(3):e17567. doi: 10.1371/journal.pone.0017567 (PMC3050922; doi:10.1371/journal.pone.0017567)
Supplement: File S1 — Robustness of our results to variation in (i) the steepness of competitive hierarchy and (ii) the maximal reproductive value (cmax) and mortality (m). (DOC) [file pone.0017567.s011.doc]

**Supplementary information file S1: Robustness of our results**

*Steepness of the local competitive hierarchy*

We tested the robustness of our main results to the steepness of the local competitive hierarchy. We used the following equation to generate the matrix *SRS* as defined in the text (equation 2).

the parameter theta is the steepness of the competitive hierarchy (that was fixed at 5 in our main simulations). We performed 2000 simulations for three different values of theta (*θ* = 5 steep competitive hierarchy, *θ* = 1 linear competitive hierarchy and *θ* = 2.5 intermediate scenario). Results are given in the Figure S3, S4, S5 and S6.

Globally, we found qualitatively similar results, illustrating the robustness of our results to different steepness of competitive hierarchy. We found that the effect of varying regional similarity was less strong when the competitive hierarchy was linear (*θ* = 1). In this case the competitive hierarchy is smooth, any deviation from SRS changes the competitive hierarchy and thus the source-sink status of the communities is less robust. This resulted in less contrasted patterns in the relationship between species richness and regional similarity as illustrated in Fig. S3 and S4.

*Variations in reproductive values and mortality*

We tested the robustness of our main results to different values of the maximal reproductive value *c_max_* (as in equation 2) and mortality (*m*). We performed 2000 simulations for three different combinations of *c_max_* and *m* *c_max_* = 5 and *m* = 0.2 as in our main simulations, *c_max_* = 2.5 and *m* = 0.2 reduced maximal reproductive rate, *c_max_* = 5 and *m* = 1 increased mortality). Results are presented in Figure S7, S8, S9 and S10.

Globally, we found very similar results, illustrating the robustness of our results to different values of *c_max_* and *m* (Fig. S7, S8, S9 and S10). We found lower regional competitive abilities when *c_max_* = 2.5 (Fig. S9) simply because competitive abilities are derived from reproductive rates (that were lower in this case). We found higher regional competitive abilities when *m* = 1 (Fig. S9) because in that case the weaker competitors are excluded from the metacommunity (because of higher mortality). The relaxation times were lower when mortality was higher (Fig. S10) but the results are qualitatively the same.
